# Eosinophils and basophils in severe fever with thrombocytopenia syndrome patients: Risk factors for predicting the prognosis on admission

**DOI:** 10.1371/journal.pntd.0010967

**Published:** 2022-12-21

**Authors:** Zishuai Liu, Rongling Zhang, Yuanni Liu, Ruize Ma, Ligang Zhang, Zhe Zhao, Ziruo Ge, Xingxiang Ren, Wei Zhang, Ling Lin, Zhihai Chen

**Affiliations:** 1 Department of Infectious Disease, Beijing Ditan Hospital, Capital Medical University, Beijing, China; 2 Department of Infectious Diseases, Yantai City Hospital for Infectious Disease, Yantai, China; Seoul National University College of Medicine, REPUBLIC OF KOREA

## Abstract

**Background:**

Severe fever with thrombocytopenia syndrome (SFTS) virus (SFTSV) is an emerging tick-borne phlebovirus with a high fatality rate. Previous studies have demonstrated the poor prognostic role of eosinophils (EOS) and basophils (BAS) in predicting multiple viral infections. This study aimed to explore the role of EOS and BAS in predicting prognosis of patients with SFTS.

**Methodology:**

A total of 194 patients with SFTS who were admitted to Yantai City Hospital from November 2019 to November 2021 were included. Patients’ demographic and clinical data were collected. According to the clinical prognosis, they were divided into survival and non-survival groups. Independent risk factors were determined by univariate and multivariate logistic regression analyses.

**Findings:**

There were 171 (88.14%) patients in the survived group and 23 (11.86%) patients in the non-survived group. Patients’ mean age was 62.39 ± 11.85 years old, and the proportion of males was 52.1%. Older age, neurological manifestations, hemorrhage, chemosis, and increased levels of laboratory variables, such as EOS% and BAS% on admission, were found in the non-survival group compared with the survival group. EOS%, BAS%, aspartate aminotransferase (AST), direct bilirubin (DBIL), and older age on admission were noted as independent risk factors for poor prognosis of SFTS patients. The combination of the EOS% and BAS% had an area under the curve (AUC) of (0.82; 95% CI: 0.725, 0.932, *P* = 0.000), which showed an excellent performance in predicting prognosis of patients with SFTS compared with neutrophil-to-lymphocyte ratio (NLR), and both exhibited a satisfactory performance in predicting poor prognosis compared with De-Ritis ratio (AST/alanine aminotransferase (ALT) ratio). EOS% and BAS% were positively correlated with various biomarkers of tissue damage and the incidence of neurological complications in SFTS patients.

**Conclusion:**

EOS% and BAS% are effective predictors of poor prognosis of patients with early-stage SFTS. The combination of EOS% and BAS% was found as the most effective approach.

## Author summary

Severe fever with thrombocytopenia syndrome (SFTS) is an emerging tick-borne infectious disease caused by a novel phlebovirus (SFTS virus, SFTSV), which has a wide range of clinical manifestations, from hyperthermia, thrombocytopenia, leukopenia, and gastrointestinal symptoms to hemorrhage, altered consciousness, and multiple organ dysfunction. It has a high mortality rate of about 11–30%. There is no specific treatment for SFTS, thus it is urgent to concentrate on patients infected with SFTSV and to identify the associated risk factors to reduce the number of critically ill and fatal cases. In the present study, EOS% and BAS% were for the first time used as variables to predict clinical outcomes of early-stage SFTS patients. The combination of EOS% and BAS% exhibited a satisfactory predictive performance compared with previously reported measures related to clinical outcomes. We also found that EOS% and BAS% were associated with neurological symptoms and signs. This study contributed to the risk factors findings on SFTS, which will be useful as guidelines for identifying the critical patients with SFTS.

## Introduction

Severe fever with thrombocytopenia syndrome (SFTS) is an emerging tick-borne infectious disease caused by a novel phlebovirus (SFTS virus, SFTSV), which belongs to the family phenuiviridae of the order Bunyavirales [1]. SFTS was initially reported in China in 2009 [2], and from 2010 to 2018, a total of 7,721 confirmed cases of SFTS were reported in 25 provinces of China [3]. SFTS cases have also been reported in Korea [4], Japan [5], and Vietnam [6]. Moreover, the Heartland virus genotype from sufferers in the United States is comparable to SFTSV [7]. SFTSV has multiple transmission routes. A previous study found that humans are the primary host of SFTSV, and human infection primarily occurs through tick bites, leading to human-to-human transmission. [8] In addition, direct contact with the body fluids of infected animals can lead to SFTSV infection in humans, and even aerosol formation is a potential transmission route of SFTSV [9,10].

SFTS has a wide range of clinical manifestations, from hyperthermia, thrombocytopenia, leukopenia, and gastrointestinal symptoms to hemorrhage, altered consciousness, and multiple organ dysfunction. It has a high mortality rate of about 11–30%, and aging, high viral load, and neurological manifestations are risk factors associated with poor prognosis [2,5,11–13]. Due to its high lethality and potential for pandemic transmission, SFTS is classified as a nationally reported disease in China, and the World Health Organization (WHO) listed SFTS as one of the top 10 priority infectious diseases in urgent need of investigation in 2017 [14]. Although some clinical trials have shown that Favipiravir can reduce the mortality of SFTS, the experimental design of the existing studies needs further improvement to clarify its efficacy [15]. Thus, it is urgent to concentrate on patients infected with SFTSV and to identify the associated risk factors to reduce the number of critically ill and fatal cases.

Previous studies have suggested that the primary role of eosinophils (EOS) and basophils (BAS) is associated with anti-parasitic and allergic reactions. Later, antiviral effects of EOS and BAS were confirmed, which were mainly reported in severe acute respiratory syndrome coronavirus 2 (SARS-COV-2), human immunodeficiency virus (HIV), influenza A viruses, and respiratory syncytial virus (RSV) [16–22]. Our retrospective study found significant differences in EOS% and BAS% of patients who were weakened compared with those who survived. We observed the elevated EOS% and BAS% in the non-survived group compared with the survived group, which was in contrast with other viral infections. They were also positively correlated with the frequency of neurological manifestations. The present study aimed to investigate EOS% and BAS% in the differential diagnosis and prognostic assessment of patients with SFTS using routine blood tests. In addition, it was attempted to explore the underlying mechanism and to elucidate its clinical significance of SFTS.

## Methods

### Ethics statement

This research was approved by the Ethics Committee of Beijing Ditan Hospital, Capital Medical University (Beijing, China; Approval No. DTEC-KY2022-022-01), and it was conducted in accordance with the principles of the Declaration of Helsinki. Written informed consent was obtained from all participants.

### Study design and patients’ enrollment

**Fig 1:**
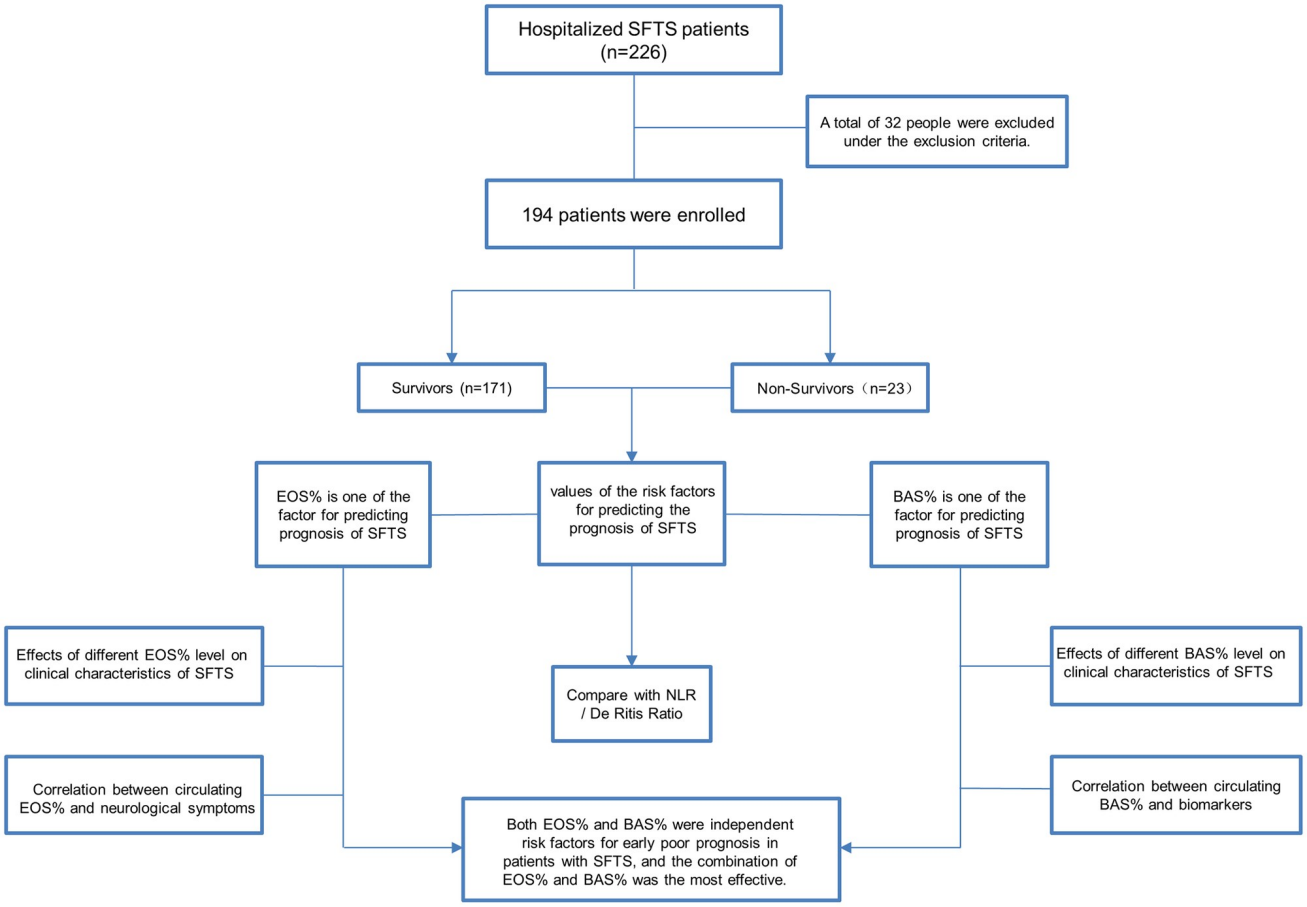
Schematic illustration of the study design.

This retrospective study included 226 SFTS patients from Yantai City Hospital (Yantai, China) between November 2019 and November 2021. The inclusion criteria were as follows: (1) Existence of epidemiological data; (2) Patients with fever (temperature >37.5°C); (3) Occurrence of thrombocytopenia; (4) Patients with positive-serum nucleic acid test, immunoglobulin G (IgG) and/or IgM antibody for SFTSV, or SFTS isolated from specimens. However, 32 patients were excluded based on the following exclusion criteria: (1) Patients with other viral infections, such as coronavirus disease 2019 (COVID-19) and hemorrhagic fever with renal syndrome (HFRS); (2) Patients with autoimmune diseases; (3) Patients with acute and chronic liver diseases; (4) Patients with blood disorders, such as leukemia and idiopathic thrombocytopenia; (5) Patients undergoing radiotherapy or chemotherapy for diverse types of cancer; (6) Patients receiving transfusion of blood products in two weeks; (7) Incomplete clinical data (Fig 1).

### Data collection

We collected patients’ demographics (gender, age, disease history, disease course, outcome), vital signs, neurological examination of the nervous system, and laboratory tests, including routine blood tests, biochemical tests, coagulation, tissue damage, and inflammatory biomarkers. The collected data were then analyzed by two professional researchers using Einmatrix platform (https://www.einmatrix.com/#!/signin) on admission.

### Definition

Neurological examination included the assessment of neurological signs and consciousness disorders. Skin change was defined as occurrence of at least one of the following signs: skin color changing, skin eruption, and nodule. Hemorrhage was defined as occurrence of at least one of the following symptoms: petechia, purpura, ecchymosis, hemoptysis, hematemesis, and melena. Neurological sign was defined as appearance of at least one of the following changes:muscle tension, involuntary movements, and neural reflexes. The observational endpoint was defined as in-hospital death or discharge on improvement.

### Statistical analysis

Normally distributed data were expressed as mean ± standard deviation (x ± s), in which they were compared between groups using the independent-samples t-test, and one-way analysis of variance (ANOVA) was utilized for making comparison among multiple groups. Abnormally distributed data were expressed as median (M) with interquartile range (IQR), in which they were compared between groups using the Mann-Whitney U test, and Kruskal-Wallis test was utilized for making comparison among multiple groups. Categorical variables were expressed as percentage (n, %) and were analyzed by the χ2 test or the Fisher’s exact test. Univariate and multivariate logistic regression analyses were performed to determine factors associated with the severity of SFTS. However, to identify independent prognostic factors for SFTS, variables with *P*-values less than 0.1 in the univariate logistic regression were imported into the multivariate logistic regression using the forward stepwise approach. Hosmer–Lemeshow test (H-L test) determined the model’s good calibration (predictive accuracy). The predictive performance of the model for in-hospital mortality in early-stage was further evaluated by the receiver operating characteristic (ROC) curve analysis. The ROC curve analysis was used to calculate the optimal cut-off values for EOS% and BAS%. Finally, correlation matrixes were generated using the Spearman correlation coefficient, which did not make any assumption about the underlying distribution. The statistical analysis was conducted using SPSS 25.0 software (IBM, Armonk, NY, USA). A two-sided P < 0.05 was considered statistically significant.

## Results

### SFTS patients’ demographics and clinical characteristics

The study included 194 patients who were admitted to the Yantai City Hospital from November 2019 to November 2021. Patients were assigned into two groups depending on clinical outcomes, including 171 (88.14%) patients in the survival group and 23 (11.86%) patients in the non-survival group. For patients who were diagnosed with SFTS, their demographic and clinical characteristics are summarized in Tables 1–3.

**Table 1:** Demographics and clinical characteristics of patients with SFTS.

Parameters	Total (n = 194)	Survival (n = 171)	Non-survival (n = 23)	*P* value
Age, years	62.39±11.85	61.20±11.38	71.22±11.76	0.000
≤45, n (%)	16/194(8.2)	15/171(8.8)	1/23(4.3)	0.749
46–60, n (%)	64/194(33.0)	62/171(36.3)	2/23(8.7)	0.008
61–75, n (%)	86/194(44.3)	75/171(43.9)	11/23(47.8)	0.719
≥76, n (%)	28/194(14.4)	19/171(11.1)	9/23(39.1)	0.001
Male, n (%)	101/194(52.1)	89/171(52.0)	12/23(52.2)	0.991
Time from onset to admission, days	5.0(4.0–7.0)	5.0(4.0–7.0)	5.0(4.0–7.0)	0.954
≤3, n (%)	42/194(21.6)	38/171(22.2)	4/23(17.4)	0.796
4–7, n (%)	114/194(58.8)	100/171(58.5)	14/23(60.9)	0.827
>7, n (%)	38/194(19.6)	33/171(19.3)	5/23(21.7)	1.000
Hospitalization, days	10.0(6.0–13.0)	10.0(7.0–13.0)	4.0(2.0–6.0)	0.000
≤7, n (%)	67/194(34.5)	47/171(27.5)	20/23(87.0)	0.000
8–14, n (%)	93/194(47.9)	90/171(52.6)	3/23(13.0)	0.000
>14, n (%)	34/194(17.5)	34/171(19.9)	0	0.039
Highest body temperature, °C	38.0(37.0–38.8)	38.0(37.0–38.8)	37.8(36.8–38.6)	0.440
38–38.9°C, n (%)	60/194(30.9)	53/171(31.0)	7/23(30.4)	0.957
>39°C, n (%)	40/194(20.6)	36/171(21.1)	4/23(17.4)	0.894
Bite by ticks, n (%)				
Positive	40/194(20.6)	39/171(22.8)	1/23(4.3)	0.075
Negative	128/194(66.0)	107/171(62.6)	21/23(91.3)	0.006
Unkown	26/194(13.4)	25/171(14.6)	1/23(4.3)	0.302
History, n (%)	78/194(40.2)	71/171(41.5)	7/23(30.4)	0.309
Diabetes	7/194(3.6)	7/171(4.1)	0	1.000
Hypertensive disease	31/194(16.0)	28/171(16.4)	3/23(13.0)	0.915
CHD	11/194(5.7)	9/171(5.3)	2/23(8.7)	0.851
Cerebral infarction	9/194(4.6)	9/171(5.3)	0	0.549
Liver disease	6/194(3.1)	5/171(2.9)	1/23(4.3)	0.536
Tumor related history	4/194(2.1)	4/171(2.3)	0	1.000
kidney disease	1/194(0.5)	1/171(0.6)	0	1.000
Other medical histories	27/194(13.9)	24/171(14.0)	3/23(13.0)	1.000

Abbreviations: CHD: Coronary Heart Disease

Continuous variable data are presented as mean (SD), median(interquartile ranges, IQR). Classified variable dates are presented as n/N (%), where N is the total number of patients with available data. *P* values comparing the group of survival and the group of non-survival.

**Table 2:** Symptomatic and signs characteristics of patients with SFTSV infection on admission.

Parameters	Total (n = 194)	Survival (n = 171)	Non-survival (n = 23)	*P* value
**Symptoms, n (%)**				
Fever	127/194(65.5)	112/171(65.5)	15/23(65.2)	0.545
Shiver	68/194(35.1)	62/171(36.3)	6/23(26.1)	0.337
Weak	124/194(63.9)	110/171(64.3)	14/23(60.9)	0.746
Chest distress	6/194(3.1)	5/171(2.9)	1/23(4.3)	0.536
Palpitation	1/194(0.5)	1/171(0.6)	0	1.000
Muscular soreness	81/194(41.8)	73/171(42.7)	8/23(34.8)	0.470
Arthralgia	47/194(23.2)	44/171(25.7)	3/23(9.4)	0.044
Oliguria	7/194(3.6)	7/171(4.1)	0	1.000
Digestive system Symptoms	167/194(86.1)	149/171(87.1)	18/23(78.3)	0.405
Inappetence	145/194(74.7)	132/171(77.2)	13/23(56.5)	0.032
Nausea	96/194(49.5)	89/171(52.0)	7/23(30.4)	0.052
Vomiting	31/194(16.0)	27/171(15.8)	4/23(17.4)	1.000
Bloating	13/194(6.7)	12/171(7.0)	1/23(4.3)	0.971
Abdominal pain	15/194(7.7)	14/171(8.2)	1/23(4.3)	0.817
Diarrhea	26/194(13.4)	21/171(12.3)	5/23(21.7)	0.355
Neurological Symptoms	23/194(11.9)	15/171(8.8)	8/23(34.8)	0.001
Confusion	14/194(7.2)	10/171(5.8)	4/23(17.4)	0.114
Delirium	1/194(0.5%)	0	1/23(4.3)	0.119
Stupor	5/194(2.)	5/171(2.9)	0	1.000
Somnolence	1/194(0.5)	0	1/23(4.3)	0.119
Coma	2/194(1.0)	0	2/23(8.7)	0.014
**Signs, n (%)**				
Skin changes	24/194(12.4)	18/171(10.5)	6/23(26.1)	0.073
Hemorrhage	6/194(3.1)	2/171(1.2)	4/23(17.4)	0.002
Pharyngeal swelling	43/194(22.2)	37/171(21.6)	6/23(26.1)	0.630
Chemosis	10/194(5.2)	3/171(1.8)	7/23(30.4)	0.000
Breath rough	36/194(18.6)	30/171(17.5)	6/23(26.1)	0.482
Rales	12/194(6.2)	8/171(4.7)	4/23(17.4)	0.055
Lymphadenectasis	29/194(14.9)	24/171(14.0)	5/23(21.7)	0.508
Hepatosplenomegaly	15/194(7.7)	15/171(8.8)	0	0.288
Abdominal Tenderness	15/194(7.7)	13/171(7.6)	2/23(8.7)	1.000
Hyperactive bowel sounds	3/194(1.5)	3/171(1.18)	0	1.000
Neurological signs	25/194(12.9)	16/171(9.4)	9/23(39.1)	0.000

Classified variable dates are presented as n/N (%), where N is the total number of patients with available data. *P* values comparing the group of survival and the group of non-survival.

**Table 3:** Laboratory results of patients with SFTS on admission.

Parameters (reference values)	Total (n = 194)	Survival (n = 171)	Non-survival (n = 23)	*P* value
Blood routine				
WBC (3.5–9.5×10^9^/L)	2.39 (1.50–4.06)	2.38 (1.51–3.86)	2.85 (1.36–4.25)	0.662
<3.5, n (%)	135/194(69.6)	121/171(70.8)	14/23(60.9)	0.333
>9.5, n (%)	5/194(2.6)	3/171(1.8)	2/23(8.7)	0.204
Neutrophils (1.8–6.3×10^9^/L)	1.31 (0.80–2.48)	1.24 (0.79–2.44)	2.02 (0.91–3.34)	0.293
<1.8, n (%)	117/194(60.3)	106/171(62.0)	11/23(47.8)	0.192
>6.3, n (%)	6/194(3.1)	4/171(2.3)	2/23(8.7)	0.312
NEU% (40–75)	61.85 (46.94–74.46)	59.70 (46.94–73.74)	69.90 (44.80–77.90)	0.363
<40, n (%)	26/194(13.4)	23/171(13.5)	3/23(13.0)	1.000
>75, n (%)	46/194(23.7)	38/171(22.2)	8/23(34.8)	0.184
Lymphocytes (1.1–3.2×10^9^/L)	0.62 (0.38–1.15)	0.63 (0.39–1.21)	0.44 (0.31–0.80)	0.079
<1.1, n (%)	139/194(71.6)	120/171(70.2)	19/23(82.6)	0.214
>3.2, n (%)	5/194(2.9)	0	5/23()	0.528
LYM% (20–50)	29.62 (16.99–42.64)	30.30 (19.20–43.54)	19.40 (14.80–33.80)	0.017
<20, n (%)	59/194(30.4)	47/171(27.5)	12/23(52.2)	0.016
>50, n (%)	20/194(10.3)	20/171(11.6)	0	0.071
Monocytes (0.1–0.6×10^9^/L)	0.15 (0.07–0.36)	0.15 (0.07–0.33)	0.16 (0.03–0.56)	0.682
<0.1, n (%)	70/194(36.1)	60/171(35.1)	10/23(43.5)	0.431
>0.6, n (%)	24/194(12.4)	20/171(11.7)	4/23(17.4)	0.659
MON% (3–10)	6.50 (3.64–10.47)	6.50 (3.80–9.84)	7.40 (1.60–27.00)	0.669
<3, n (%)	34/194(17.5)	26/171(15.2)	8/23(34.8)	0.043
>10, n (%)	109/194(56.2)	104/171(60.8)	5/23(51.7)	0.000
Eosinophils (0.02–0.5×10^9^/L)	0 (0–0.01)	0 (0–0.01)	0.01 (0–0.15)	0.010
<0.02, n (%)	164/194(84.5)	150/171(87.7)	14/23(60.9)	0.002
>0.5, n (%)	4/194(2.1)	1/171(0.6)	3/23(13.0)	0.002
EOS% (0.4–8)	0 (0–0.4)	0 (0–0.30)	0.50 (0–1.90)	0.000
<0.4, n (%)	143/194(73.7)	134/171(78.4)	9/23(39.1)	0.000
0.4–8, n (%)	51/194(26.3)	37/171(21.6)	14/23(60.9)	0.000
Basophils (0–0.06×10^9^/L)	0 (0–0.01)	0 (0–0.01)	0 (0–0.03)	0.079
BAS% (0–1)	0.10 (0–0.40)	0.04 (0–0.40)	0.30 (0.20–0.90)	0.000
RBC (4.3–5.8×10^9^/L)	4.49 (4.14–4.86)	4.48 (4.16–4.86)	4.57 (3.76–5.04)	0.584
HGB (130-175g/L)	137.50 (127.00–149.00)	137.00 (127.00–147.00)	148.00 (126.00–155.00)	0.198
PLT (125–350×10^9^/L)	57.50 (40.00–78.75)	59.00 (41.00–84.00)	39.00 (18.00–59.00)	0.000
MPV (6.5–12 fL)	11.20 (10.28–12.00)	11.30 (10.50–12.20)	10.0 (8.60–11.00)	0.000
Tissue damage marker				
LDH (80–285 U/L)	668.0(392.00–1076.50)	573.4(375.00–922.00)	900.0(682.00–2506.35)	0.000
CK (0–190 U/L)	374.5(170.00–997.00)	326.0(145.00–890.12)	1655.9(2663.00–425.90)	0.000
Biochemical marker				
ALT (9–50 U/L)	89.00 (49.00–165.56)	80.53 (47.00–136.00)	165.10 (109.00–283.00)	0.000
AST (15–40 U/L)	160.15 (80.33–323.93)	153.00 (71.00–267.00)	645.0(247.00–1082.00)	0.000
TBIL (2.0–20.4 umol/L)	10.20 (7.79–14.03)	10.10 (7.40–13.50)	15.10 (9.00–23.70)	0.002
DBIL (0–6.8 umol/L)	4.65 (3.40–6.30)	4.50 (3.20–6.08)	6.89 (5.06–12.42)	0.000
ALB (35–53 g/L)	33.37 (30.00–36.62)	33.70 (31.00–37.00)	27.00 (28.10–31.00)	0.000
GLOB (20–40 g/L)	24.47 (21.68–26.80)	24.41 (21.50–26.60)	24.75 (22.55–28.20)	0.193
GGT (11–49 U/L)	50.50 (24.00–96.00)	46.00 (23.00–96.00)	113.00 (65.00–195.80)	0.000
ALP (40–150 U/L)	67.00 (52.76–87.75)	65.00 (52.00–80.00)	96.00 (66.00–192.00)	0.000
GLU (4.16–6.44 mmol/L)	6.57 (5.60–8.16)	6.51 (5.58–8.16)	7.50 (5.70–9.00)	0.317
K^+^ (3.5–5.1 mmol/L)	3.84 (3.53–4.15)	3.81 (3.53–4.15)	3.90 (3.67–4.15)	0.629
Na^+^ (136–146 mmol/L)	134.00 (130.80–136.60)	134.00 (130.40–136.70)	134.00 (131.40–136.00)	0.825
Ca^2+^ (2.2–2.55 mmol/L)	1.95 (1.90–2.02)	1.95 (1.92–2.02)	1.91 (1.76–1.97)	0.009
PCT (0–0.06 ng/ml)	13.00 (11.98–14.74)	0.46 (0.11–0.81)	0.81 (0.50–1.72)	0.001
CRP (5-10mg/L)	7.69 (2.30–11.64)	6.10 (1.98–11.64)	11.64 (10.20–23.70)	0.001
Kidney injury				
UREA (1.7–8.3 mmol/L)	5.28 (4.10–7.16)	5.09 (4.07–7.13)	7.34 (5.97–11.13)	0.000
CREA (40-106umol/L)	66.28 (54.45–81.27)	64.20 (54.00–81.00)	81.27 (63.50–110.90)	0.002
Coagulation				
TT (14-21s)	19.65 (17.20–21.00)	19.65 (17.50–20.90)	18.80 (15.80–24.90)	0.763
APTT (28–43.5s)	46.90 (38.25–48.16)	46.70 (38.60–48.61)	61.30 (33.70–85.00)	0.052
PT (11–14.5s)	13.00 (11.98–14.74)	13.20 (12.00–14.74)	12.60 (11.50–13.80)	0.177
INR (0.8–1.2)	1.02 (1.00–1.09)	1.02 (1.00–1.09)	1.01 (0.95–1.10)	0.372

Abbreviations: WBC: White Blood Cell, NEU: Neutrophil, LYM: Lymphocyte, MON: Monocyte, EOS: Eosinophils, BAS: Basophils, RBC: Red Blood Cell, HGB: Hemoglobin, PLT: Platelet, MPV: Mean Platelet Volume, LDH: Lactate dehydrogenase, CK: Creatine phosphokinase, ALT: Alanine aminotransaminase, AST: Aspartate aminotransferase, TBIL: Total Bilirubin, DBIL: Direct Bilirubin, ALB: Albumin, GLOB: Globulin, GGT: γ-glutamyl transferase, ALP: Alkaline phosphatase, GLU: Glucose, PCT: Procalcitonin, CRP: C-reactive protein, CREA: Creatinine, TT: Thrombin Time, APTT: Activated Partial Thromboplastin Time, PT: Prothrombin time, INR: Internationally Standardized Ratio.

Continuous variable data are presented as median (interquartile ranges, IQR). Classified variable dates are presented as n/N (%), where N is the total number of patients with available data. *P* values comparing the group of survival and the group of non-survival.

Patients’ mean age was 62.39 ± 11.85 years old, in which patients in the non-survival group (71.22±11.76 years old) were older than those in the survival patient group (61.20±11.38 years old). Patients in the non-survival group had a shorter hospitalization than those in the survival group, in which 20 (87.0%) cases experienced shortened hospitalization. There were no significant differences between the two groups regarding gender, time from onset to admission, body temperature, history of tick bites, hypertensive disease, diabetes, coronary heart disease (CHD), and history of other diseases. Symptoms of digestive disorders (86.1%) accounted for the highest proportion, including poor appetite, nausea, vomiting, bloating, abdominal pain, and diarrhea, followed by fever (65.5%), and fatigue (63.9%). Compared with the survival group, higher incidence rates of chemosis, hemorrhage, and neurological manifestations were found in the non-survival group. A decrease in platelet (PLT) count was found in all the patients. In the non-survival group, higher levels of EOS%, BAS%, alanine aminotransferase (ALT), aspartate aminotransferase (AST), dehydrogenase (LDH), creatine kinase (CK), alkaline phosphatase (ALP), gamma-glutamyl transpeptidase (GGT), urea, creatinine (CREA), C-reactive protein (CRP), procalcitonin (PCT), and lower lymphocyte (LYM)%, mean platelet volume (MPV), Ca^2+^, PLT, and albumin (ALB) were detected compared with those in the survival patients. No significant differences were detected between the two groups for the remaining indicators.

### Independent risk factors for non-survived patients with SFTS

The independent risk factors of SFTS were explored for early and effective identification of severe SFTS patients and prediction of their clinical outcomes. Significant predictors were first selected by univariate logistic regression analysis (S1 Table). The results of multivariate logistic regression analysis could be summarized as follows: age (odds ratio (OR), 1.070; 95% confidence interval (CI): 1.007–1.137, *P* = 0.028), EOS% (OR, 3.215; 95% CI: 1.543–6.699, *P* = 0.002), BAS% (OR, 2.290; 95% CI: 1.156–4.535, *P* = 0.017), AST (OR, 1.003; 95%CI: 1.001–1.005, *P* = 0.001), and direct bilirubin (DBIL) (OR = 1.120; 95%CI: 1.004–1.248, *P* = 0.041), which could serve as independent predictors of early mortality in SFTS patients.

**Fig 2:**
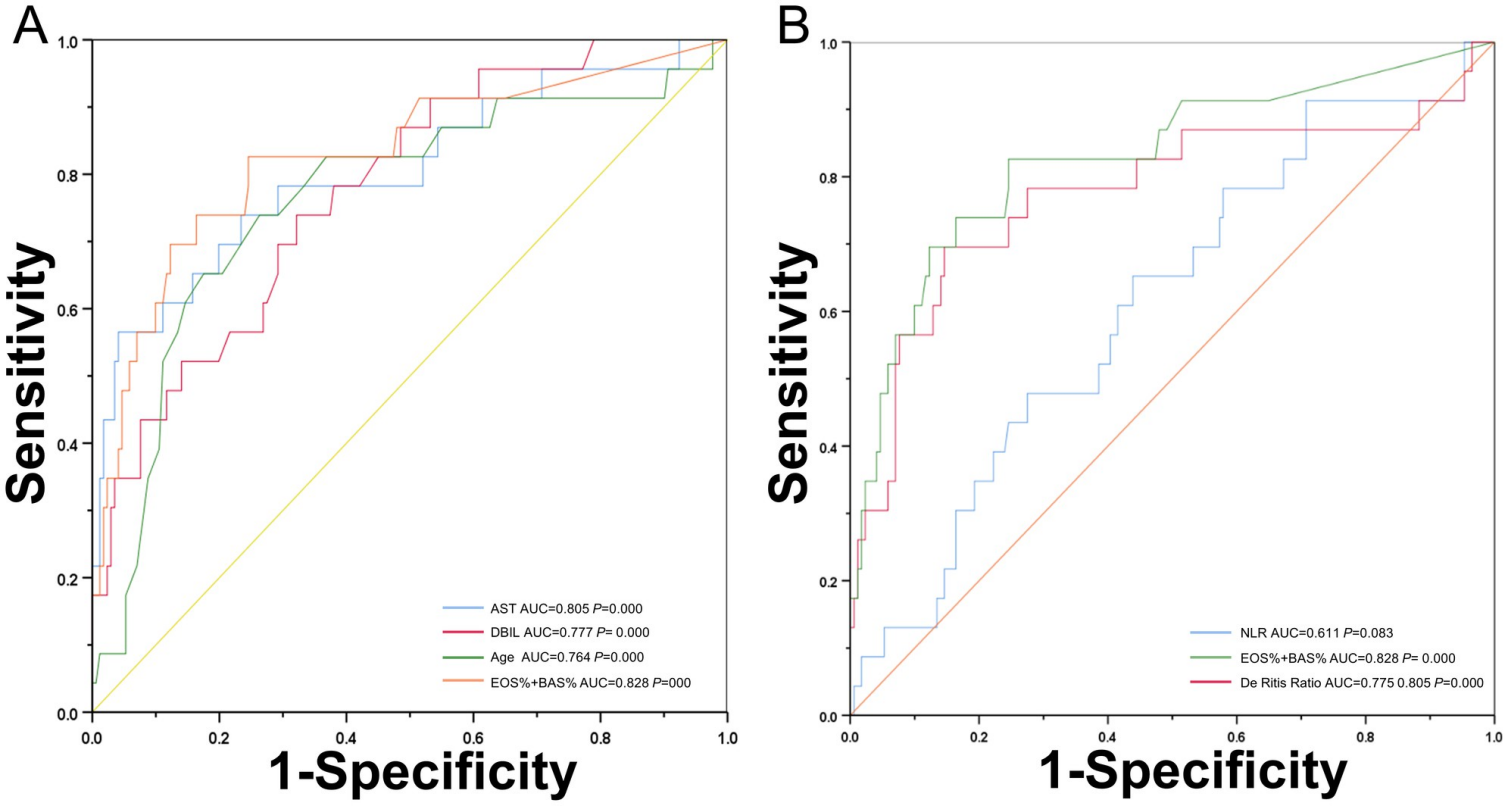
Receiver operating characteristic (ROC) curves for evaluating the predictive ability of the factors associated with severity of SFTS on admission. (A) The combination of the EOS% and BAS% (orange line) had an AUC of 0.82 (*P* = 0.000); AST (blue line) had an AUC of 0.805 (*P* = 0.000); DBIL (red line) had an AUC of 0.777 (*P* = 0.000); Age (green line) had an AUC of 0.764 (*P* = 0.000). (B) The combination of the EOS% and BAS% (green line) had an AUC of 0.828 (*P* = 0.000); De-Ritis ratio (red line) had an AUC of 0.775 and the cut-off value was 2.69 (*P* = 0.000); NLR (blue line) had an AUC of 0.611 and the cut-off value was 2.27 (*P* = 0.083). Abbreviations: EOS: Eosinophils, BAS: Basophils, AST: Aspartate aminotransferase, DBIL: Direct Bilirubin, AUC: Area under the ROC curve, CI: Confidence interval, De-Ritis ratio: AST/ALT ratio, NLR: Neutrophil-to-lymphocyte ratio.

According to the results of the multivariate logistic regression analysis, the logistic regression equation can be expressed as follows: logit(p) = -3.055+1.116EOS%+1.235BAS%. The EOS% had an area under the curve (AUC) of 0.744 (95% CI: 0.616–0.872, *P* = 0.000), BAS% had an AUC of 0.721 (95% CI: 0.614–0.828, *P* = 0.001), and the combination of the EOS% and BAS% had an AUC of 0.82 (95% CI: 0.725–0.932, *P* = 0.000), indicating a good predictive performance compared with other risk factors in our cohort (Fig 2A). The AST/ALT (De-Ritis) ratio had an AUC of 0.775 (95% CI: 0.648–0.903, *P* = 0.000) and neutrophil-to-lymphocyte ratio (NLR) had an AUC of 0.611 (95% CI: 0.491–0.732, *P* = 0.083). Optimum cutoff value was calculated from the largest Youden’s index (Table 4).

**Table 4:** Predictive value of risk factors for SFTS severity.

Parameters	AUC	Cut off Values	Sensitivity	Specificity	95%CI	*P* value
EOS%	0.744	0.35	0.652	0.784	(0.616,0.872)	0.000
BAS%	0.721	0.17	0.826	0.614	(0.614,0.828)	0.001
AST	0.805	628.30	0.565	0.959	(0.693,0.971)	0.000
DBIL	0.777	5.51	0.739	0.678	(0.680,0.874)	0.000
EOS%+BAS%	0.828	/	0.826	0.754	(0.725,0.932)	0.000
De Ritis Ratio	0.775	2.69	0.696	0.854	(0.648,0.903)	0.000
NLR	0.611	2.27	0.652	0.561	(0.491,0.732)	0.083

Abbreviations: EOS: Eosinophils, BAS: Basophil, AST: Aspartate aminotransferase, DBIL: Direct Bilirubin, AUC: Area Under the ROC Curve, CI: 95% Confidence Interval, De Ritis Ratio: AST/ALT-Ratio, NLR: Neutrophil-to-Lymphocyte Ratio.

The cut-off points were selected by maximizing the sum of sensitivity and specificity.

### The predictive value of EOS%+BAS% for the prognosis on admission

In our study, the OR value did not significantly change, either after adjusting for age, gender, body temperature, arthralgia, hemorrhage, symptoms of digestive disorders, neurological symptoms and signs, hypertensive disease, and CHD, indicating that the combination of the EOS% and BAS% was a stable risk factor for prognosis of SFTS patients (S2 Table). According to the H-L test (P = 0.294), the combination of EOS% and BAS% had an excellent predictability for prognosis of patients with SFTS compared with NLR, and both had a satisfactory performance in predicting poor prognosis compared with De-Ritis ratio (Fig 2B) (S3 Table).

### The effects of different EOS% levels on the clinical characteristics of SFTS patients

According to the results of the multivariate logistic regression model, EOS% was an independent risk factor for early death in patients with SFTS (OR, 3.215; 95% CI: 1.543–6.699). All patients were divided into EOS%^low^ and EOS%^high^ groups based on the cutoff value (0.35%). The EOS%^high^ group had a higher fatality rate (28.8% vs. 5.6%, *P* = 0.000), a higher percentage of hospitalization ≤7 days (48.1% vs. 29.6% *P* = 0.016), and included more patients with neurological signs (21.2% vs. 9.9%, *P* = 0.038) compared with the EOS%^low^ group. No significant differences were found between the two groups in terms of age (*P* = 0.256), gender (*P* = 0.981), the highest body temperature (*P* = 0.853), and history of the tick bite (*P* = 0.136) (Table 5).

**Table 5:** Clinical characteristics of patients with SFTS, according the EOS% cutoff value on admission.

Parameters	Total (n = 194)	EOS%^low^ (<0.35 = (n = 142)	EOS%^high^ (≥0.35) (n = 52)	*P* value
Clinic outcome, n (%)	23/194(11.84)	8/142(5.6)	15/52(28.8)	0.000
Age, years	62.39±11.85	61.74±11.03	64.17±13.80	0.256
≤45, n (%)	16/194(8.2)	13/142(9.2)	3/52(5.8)	0.642
46–60, n (%)	64/194(33.0)	48/142(33.8)	16/52(30.8)	0.691
61–75, n (%)	86/194(44.3)	67/142(47.2)	19/52(36.5)	0.186
≥76, n (%)	28/194(14.4)	14/142(9.9)	14/52(26.9)	0.003
Male, n (%)	101/194(52.1)	74/142(52.1)	27/52(51.9)	0.981
Time from onset to admission, days	5.0(4.0–7.0)	5.0(4.0–7.0)	5.0(4.0–7.5)	0.609
≤3, n (%)	42/194(21.6)	31/142(21.8)	11/52(21.2)	0.919
4–7, n (%)	114/194(58.8)	86/142(60.6)	28/52(53.8)	0.400
>7, n (%)	38/194(19.6)	25/142(17.6)	13/52(25.0)	0.250
Hospitalization, days	10.0(6.0–13.0)	7.0(10.0–13.0)	8.0(4.0–12.0)	0.008
≤7, n (%)	67/194(34.5)	42/142(29.6)	25/52(48.1)	0.016
8–14, n (%)	93/194(47.9)	73/142(51.4)	20/52(38.5)	0.110
>14, n (%)	34/194(17.5)	27/142(19.0)	7/52(13.5)	0.368
Highest body temperature, °C	38.0(37.0–38.8)	38.0(37.0–38.8)	37.9(37.0–38.6)	0.853
38–38.9°C, n (%)	60/194(30.9)	45/142(31.7)	15/52(28.8)	0.704
>39°C, n (%)	40/194(20.6)	29/142(20.4)	11/52(21.2)	0.911
Bite by ticks, n (%)	40/194(20.6)	33/142(23.2)	7/52(13.5%)	0.136
Neurological Symptoms, n (%)	23/194(11.9)	13/142(9.2)	10/52(19.)	0.054
Confusion	14/194(7.2)	7/142(4.9)	7/52(13.5)	0.085
Delirium	1/194(0.5)	0	1/52(1.9)	0.268
Stupor	5/194(2.6)	4/142(2.8)	1/52(1.9)	1.000
Somnolence	1/194(0.5)	0	1/52(1.9)	0.268
Coma	2/194(1.0)	2/142(1.4)	0	1.000
Neurological signs, n (%)	25/194(12.9)	14/142(9.9)	11/52(21.2)	0.038

Continuous variable data are presented as median (interquartile ranges, IQR).

Classified variable dates are presented as n/N (%), where N is the total number of patients with available data. *P* values comparing between the group of EOS%^low^ and the group of EOS%^high^.

Based on EOS count, patients were assigned into three groups: less than the lower limit of normal (group A), normal (group B), and greater than the upper limit of normal (group C). Group C was associated with a higher rate of death, older age, a shorter hospitalization, and a higher incidence of neurological symptoms and the presence of neurological signs than other two groups. There were no significant differences among the three groups in terms of gender (*P* = 0.369), maximum body temperature (*P* = 0.943), or history of the tick bite (*P* = 0.072) (S4 Table).

The absolute value of EOS was divided into >0 group and equal to 0 group. There were no significant differences in clinical outcomes (*P* = 0.076), age (*P* = 0.181), gender (*P* = 0.746), length of hospitalization (*P* = 0.133), the highest body temperature (*P* = 0.910), neurological symptoms (*P* = 0.076), and neurological signs (*P* = 0.066) between the two groups (S5 Table).

### Correlation between circulating EOS% and the frequency of neurological manifestations in SFTS patients

Through Spearman correlation analysis, it was revealed that EOS% was positively correlated with the frequency of neurological symptoms in SFTS patients (r = 0.158, *P* = 0.028) and was positively correlated with the frequency of neurological signs (r = 0.180, *P* = 0.012) (S6 Table).

### The effects of different BAS% levels on the clinical characteristics of SFTS patients

BAS% was found as an independent risk factor for patients with early-stage SFTS (OR, 2.290; 95% CI: 1.156–4.535, *P* = 0.017). All patients were divided into BAS%^low^ and BAS%^hight^ groups based on the cutoff value (0.17%). The BAS%^high^ group had a higher fatality rate (22.4% vs. 3.7%, *P* = 0.000), older age (65.60±11.09 vs. 59.89±11.87, *P* = 0.001), and a shorter hospitalization (9.0 days, IQR: 4.5–12.5 vs. 11.0 days, IQR: 6.0–13.0, *P* = 0.012) than the BAS%^low^ group. No significant differences were found between the two groups in terms of gender (*P* = 0.051), the highest body temperature (*P* = 0.065), history of the tick bite (*P* = 0.207), neurological symptoms (*P* = 0.680), and neurological signs (*P* = 0.394) (Table 6).

**Table 6:** Clinical characteristics of patients with SFTS, according to the BAS% cutoff value on admission.

Parameters	Total (n = 194)	BAS%^low^ (<0.17) (n = 109)	BAS%^high^ (≥0.17) (n = 85)	*P* value
Clinic outcome, n (%)	23/194(11.84)	4/109(3.7)	19/85(22.4)	0.000
Age, years	62.39±11.85	59.89±11.87	65.60±11.09	0.001
≤45, n (%)	16/194(8.2)	12/109(11.0)	4/85(4.7)	0.113
46–60, n (%)	64/194(33.0)	44/109(40.4)	20/85(23.5)	0.013
61–75, n (%)	86/194(44.3)	41/109(37.6)	45/85(52.9)	0.033
≥76, n (%)	28/194(14.4)	12/109(11.0)	16/85(18.8)	0.124
Male, n (%)	101/194(52.1)	50/109(45.9)	51/85(60.0)	0.051
Time from onset to admission, days	5.0(4.0–7.0)	5.0(3.5–7.0)	5.0(4.0–8.0)	0.102
≤3, n (%)	42/194(21.6)	27/109(24.8)	15/85(17.6)	0.232
4–7, n (%)	114/194(58.8)	67/109(61.5)	47/85(55.3)	0.386
>7, n (%)	38/194(19.6)	15/109(13.8)	23/85(27.1)	0.021
Hospitalization, days	10.0(6.0–13.0)	11.0(6.0–13.0)	9.0(4.5–12.5)	0.012
≤7, n (%)	67/194(34.5)	32/109(29.4)	35/85(41.2)	0.086
8–14, n (%)	93/194(47.9)	52/109(47.7)	41/85(48.2)	0.942
>14, n (%)	34/194(17.5)	25/109(22.9)	9/85(10.6)	0.025
Highest body temperature, °C	38.0(37.0–38.8)	38.0(37.2–38.9)	37.8(36.8–38.6)	0.065
38–38.9°C, n (%)	60/194(30.9)	34/109(31.2)	26/85(30.6)	0.928
>39°C, n (%)	40/194(20.6)	25/109(22.9)	15/85(17.6)	0.366
Bite by ticks, n (%)	40/194(20.6)	26/109(23.9)	14/85(16.5)	0.207
Neurological Symptoms, n (%)	23/194(11.9)	12/109(11.0)	11/85(12.9)	0.680
Confusion, n (%)	14/194(7.2)	7/109(6.4)	7/85(8.2)	0.628
Delirium, n (%)	1/194(0.5)	0	1/85(1.2)	0.438
Stupor, n (%)	5/194(2.6)	5/109(4.6)	0	0.123
Somnolence, n (%)	1/194(0.5)	0	1/85(1.2)	0.438
Coma, n (%)	2/194(1.0)	0	2/85(2.4)	0.191
Neurological signs, n (%)	25/194(12.9)	12/109(11.0)	13/85(15.1)	0.394

Continuous variable data are presented as median (interquartile ranges, IQR).

Classified variable date are presented as n/N (%), where N is the total number of patients with available data. *P* values comparing the group of BAS%^low^ and the group of BAS%^high^.

According to basophilic count, patients were divided into two groups: normal range and greater than the upper limit of normal. The group of greater than the upper limit of normal was associated with a higher rate of death (50.0% vs. 10.1%, *P* = 0.007) and a higher incidence of neurological symptoms (50% vs. 10.2%, *P* = 0.008) and the presence of neurological signs (50% vs. 11.3%, *P* = 0.008) than the normal range group. No significant differences were found between the two groups in age (*P* = 0.145), gender (*p* = 0.890), the highest body temperature (*P* = 0.979), or history of the tick bite (*P* = 0.305) (S7 Table).

### Correlation between circulating BAS% and clinical parameters of SFTS patients

**Fig 3:**
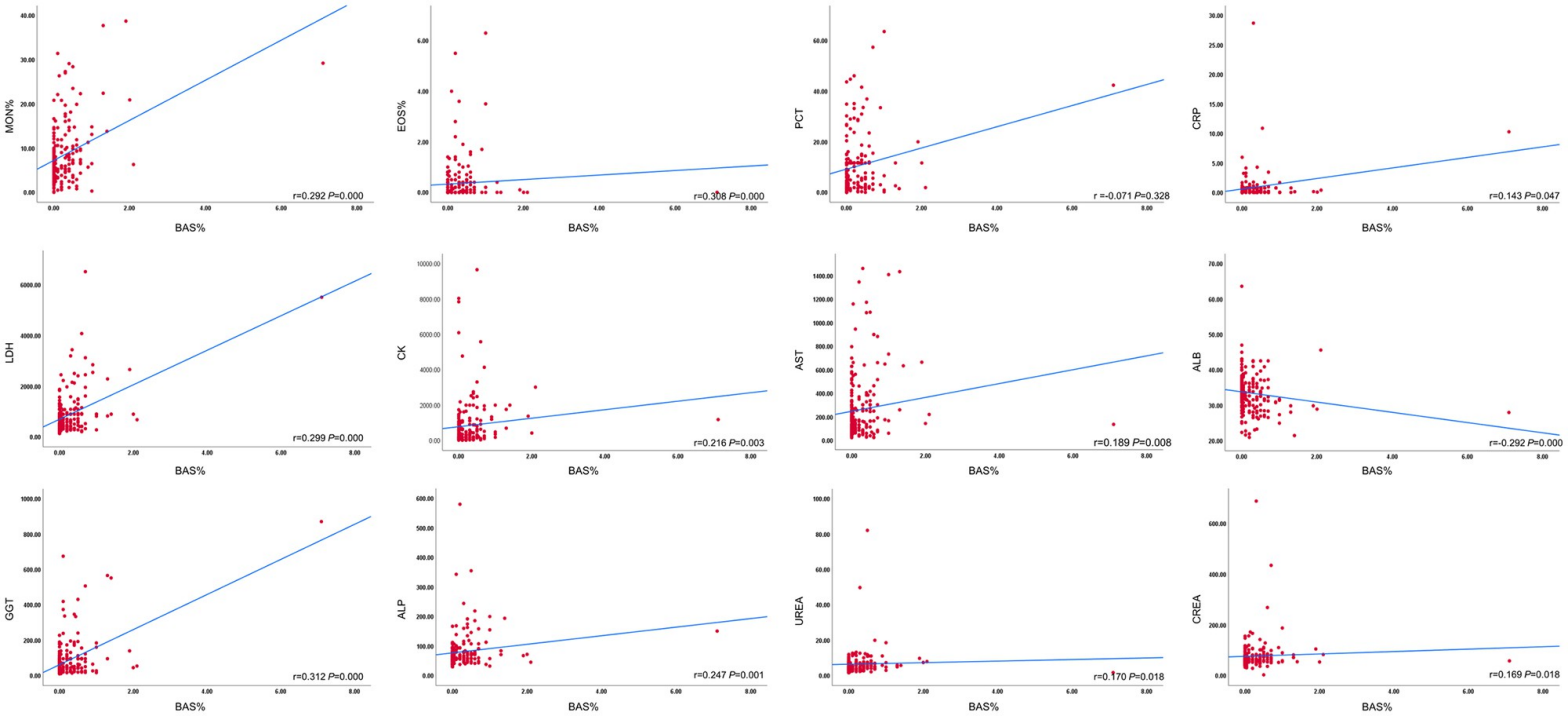
Correlation among circulating EOS%, BAS%, and laboratory parameters in SFTS patients. Abbreviations: MON: Monocyte, EOS: Eosinophils, BAS: Basophils, PCT: Procalcitonin, CRP: C-reactive protein, LDH: Lactate dehydrogenase, CK: Creatine phosphokinase, AST: Aspartate aminotransferase, ALB: Albumin, GGT: γ-glutamyl transferase, ALP: Alkaline phosphatase, CREA: Creatinine.

Through Spearman correlation analysis, it was revealed that BAS% was positively correlated with monocyte (MON)% (r = 0.292, *P* = 0.000), EOS% (r = 0.308, *P* = 0.000), LDH (r = 0.39, *P* = 0.000), CK (r = 0.216, *P* = 003), AST (r = 0.189, *P* = 0.008), ALT (r = 0.185, *P* = 0.010), total bilirubin (TBIL) (r = 0.220, *P* = 0.002), DBIL (r = 0.284, *P* = 0.000), GGT (r = 0.312, *P* = 0.000), ALP (r = 0.247, *P* = 0.001), UREA (r = 0.170, *P* = 0.018), CREA (r = 0.169, *P* = 0.018), CRP (r = 0.143, *P* = 0.047), and was negatively correlated with neutrophil (NEU)% (r = -0.142, *P* = 0.048), MPV (r = 0.174, *P* = 0.015), ALB (r = 0.292, *P* = 0.000), and PCT (r = -0.237, *P* = 0.001) (Fig 3). BAS% was positively correlated with the frequency of neurological signs in SFTS patients (r = 0.146, *P* = 0.043) (S8 Table)

## Discussion

In the present study, EOS% and BAS% were for the first time used as variables to predict clinical outcomes of early-stage SFTS patients. The combination of EOS% and BAS% exhibited a satisfactory predictive performance compared with previously reported measures related to clinical outcomes. We also found that EOS% and BAS% were associated with neurological symptoms and signs. Patients mainly presented with thrombocytopenia, liver dysfunction, elevated biomarkers of tissue damage, and a higher frequency of neurological-related manifestations, particularly in non-survived patients, which is in parallel with previous studies [3],[23,24].

EOS are bone marrow-derived leukocytes. As research has progressed, a comprehensive understanding of the critical role of EOS in immunity and host defense has emerged [20,25]. EOS has been used as an indicator for disease progression and outcomes. In our cohort, EOS% was noted as an independent risk factor for death on admission and was positively associated with neurological signs and/or symptoms. Eosinophilic Cationic Protein (ECP) is one of the main components of EOS, and the level of ECP was reported to be positively correlated with EOS% and was associated with neurological damage [26,27]. ECP can alter the permeability of cell membranes, subsequently causing calcium influx, which can ultimately lead to cell apoptosis.[28] In addition, Peng et al. showed that the increased intracellular cation levels lead to the sequential activation of the caspase-9, pro-caspase-3 and 8, inducing apoptosis of neuronal cells [29]. In our study, EOS% was found to be positively correlated with the incidence of neurological symptoms and signs, with no significant correlation with delirium, stupor, somnolence, and coma, which could be related to the inadequate number of cases with associated symptoms.

Basophils are an essential component of innate immunity and are also a promoter of type 2 immune responses, which play a role in parasitic infections, allergic reactions, and viral infections. In our study, BAS% was identified as a predictor of poor prognosis of early-stage SFTS patients, which was consistent with studies on the COVID-19 [30,31]. However, indifferent to COVID-19 [32], the relative elevation of basophils in the non-survived group compared with that in the survived group may be related to tick bite transmission.[33] With multiple pattern recognition receptors on basophils, such as Dendritic Cell-Specific Intercellular adhesion molecule 3-Grabbing Nonintegrin (DC-SIGN) and C-type lectin, basophils may provide a stable cellular basis for HIV capture and transmission [21,34]. Importantly, several studies found that SFTS enters host cells via these two receptors, thereby involving basophils as one of the target cells for SFTS [35,36]. Studies have shown that SFTSV infection drove macrophage differentiation skewed to M2 phenotype, which facilitated virus shedding, and resulted in viral spread [37]. Interleukin-4 (IL-4) production from basophils can contribute to the differentiation of macrophages towards the M2 phenotype. [38] In addition to promoting M2 macrophage differentiation, IL-4 can cause microvascular infiltration and a procoagulant state through remodeling and upregulation of the expression levels of vascular cell adhesion molecule-1 (VCAM-1) and monocyte chemoattractant protein-1 (MCP-1), which may result in damage to the endothelium [39]. IL-4 induces T cell differentiation towards the TH2 phenotype, and a significant correlation of Th1/Th2 with disease severity in SFTS patients was reported [40,41]. In addition, the activation of BAS may cause the release of large amounts of cytokines, such as IL-6 and IL-8, which are essential components of the cytokine storm and are associated with the poor prognosis of SFTS patients [42,43]. Hence, we hypothesized that organ failure in SFTS patients could be attributed to immune dysfunction associated with the involvement of BAS.

Prediction of the clinical outcomes by innate immune cells and immune checkpoints has been frequently reported. Studies have shown that immune checkpoints are associated with viral escape from host immunity [44,45]. In the study of COVID-19, programmed death-ligand 1 (PD-L1), one of the immune checkpoints, was highly expressed in EOS and BAS in severe patients, and it was positively correlated with sequential organ failure assessment (SOFA) scores, providing a new idea for subsequent studies on SFTS [46].

There are still some limitations in this study. Firstly, the small sample size should be noted, as well as the lack of viral load data, and there was no validation cohort. Secondly, the cerebrospinal fluid of patients was not examined to assess the cause of neurological symptoms because of thrombocytopenia. Finally, the role of EOS and BAS in systemic tissue damage in SFTS patients was not investigated. Hence, it is essential to eliminate the abovementioned limitations.

In conclusion, both EOS% and BAS% were found as independent risk factors for poor prognosis of patients with early-stage SFTS, and combination of EOS% and BAS% was the most effective approach. EOS% and BAS% are rapid, simple, effective, and inexpensive prognostic markers, and they may be efficacious for diagnosing and treating a variety of diseases.

## Supporting information

S1 Fig EOS% and BAS% were elevated in the non-survival group compared to the survival group and were positively correlated with the incidence of neurological complications, and their combination was highly predictive of the prognosis of SFTS patients. (Created with BioRender.com).
(TIF)

S1 Table Risk factors associated with disease prognosis of patients with SFTS. (DOCX)

S2 Table The predictive value of EOS%+BAS% for the prognosis on admission. (DOCX)

S3 Table Differences between AUC of EOS%+BAS% and other factors AUC. (DOCX)

S4 Table Clinical characteristics of patients with SFTS, according to the EOS level on admission. (DOCX)

S5 Table Clinical characteristics of patients with SFTS, according to the EOS whether decreased to Undetectable on admission. (DOCX)

S6 Table Correlation between circulating EOS%, BAS% and neurological manifestations in SFTS patients. (DOCX)

S7 Table Clinical characteristics of patients with SFTS, according to the BAS level on admission. (DOCX)

S8 Table Correlation between circulating EOS%, BAS% and laboratory paraments of SFTS patients. (DOCX)
