# Supplementary figures and images for: Eosinophils and basophils in severe fever with thrombocytopenia syndrome patients: Risk factors for predicting the prognosis on admission
Source: PLoS Negl Trop Dis. 2022 Dec 21;16(12):e0010967. doi: 10.1371/journal.pntd.0010967 (PMC9770358; doi:10.1371/journal.pntd.0010967)

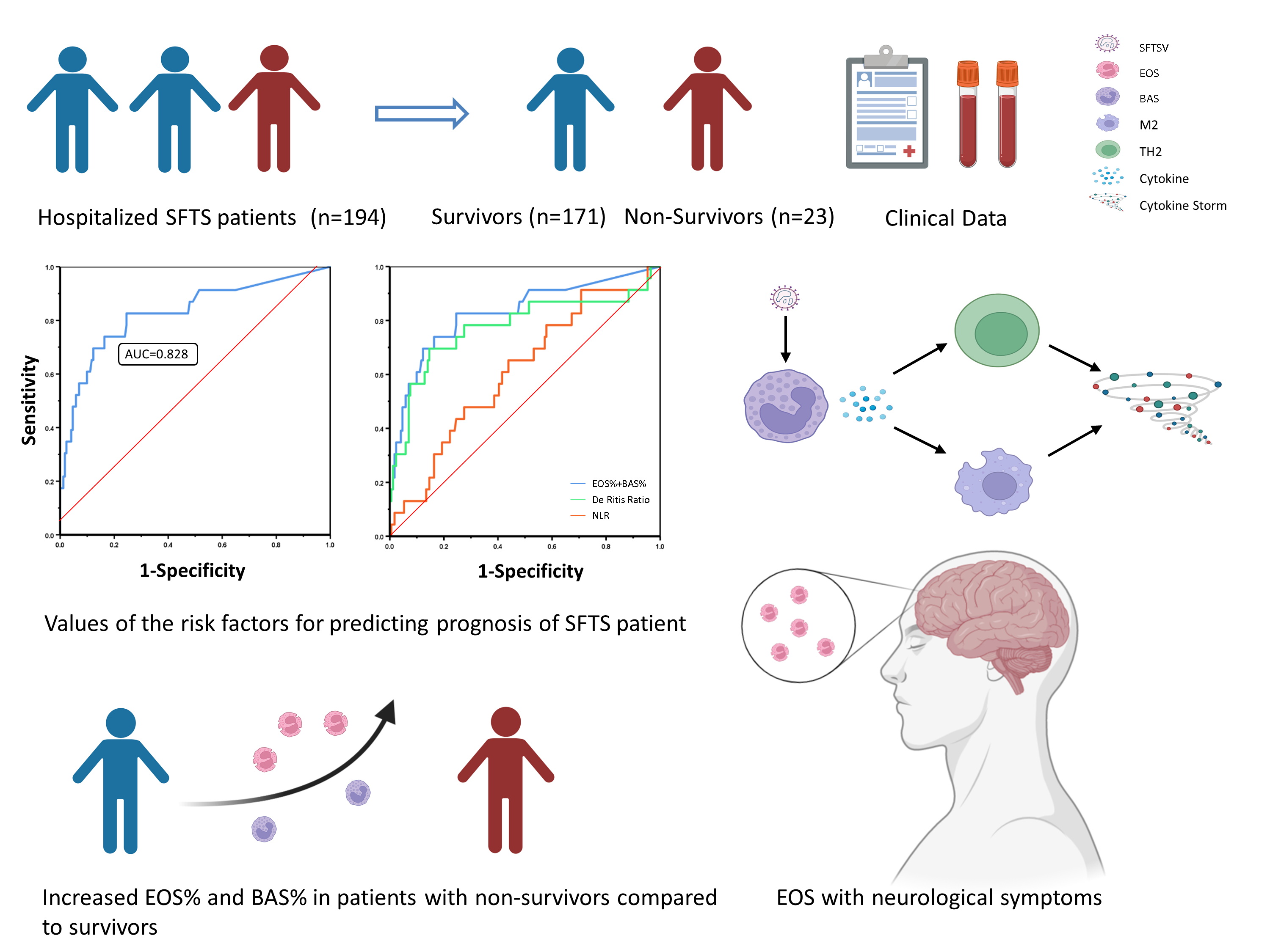

Supplement: S1 Fig — (Created with BioRender.com). (TIF) [file pntd.0010967.s001.tif]
