# Supplementary material for: Eosinophils and basophils in severe fever with thrombocytopenia syndrome patients: Risk factors for predicting the prognosis on admission
Source: PLoS Negl Trop Dis. 2022 Dec 21;16(12):e0010967. doi: 10.1371/journal.pntd.0010967 (PMC9770358; doi:10.1371/journal.pntd.0010967)
Supplement: S1 Table — (DOCX) [file pntd.0010967.s002.docx]

**S1 Table. Risk factors associated with disease prognosis of patients with SFTS**

| **Parameters** | **Univariate** | | **Multivariate** | |
| --- | --- | --- | --- | --- |
|  | **OR (95% CI)** | ***P* value** | **OR (95% CI)** | ***P* value** |
| **Demographics and baseline characters** | | | | |
| Age | 1.094(1.042-1.148) | 0.000 | 1.070(1.007-1.137) | 0.028 |
| Sex | 0.995(0.416-2.378) | 0.991 |  |  |
| Temperature | 0.844(0.553-1.289) | 0.432 |  |  |
| History | 0.616(0.241-1.576) | 0.312 |  |  |
| Hypertensive disease | 0.766(0.213-2.753) | 0.683 |  |  |
| CHD | 1.714(0.347-8.476) | 0.509 |  |  |
| Symptoms &Signs |  |  |  |  |
| Arthralgia | 0.433(0.123-1.528) | 0.193 |  |  |
| Hemorrhage | 17.789(3.053-103.647) | 0.001 |  |  |
| Digestive symptoms | 0.532(0.179-1.577) | 0.255 |  |  |
| Neurological symptoms | 5.547(2.023-15.206) | 0.001 |  |  |
| Neurological signs | 6.228(2.331-16.642) | 0.000 |  |  |
| **Laboratory findings** |  |  |  |  |
| WBC | 1.098(0.930-1.296) | 0.268 |  |  |
| NEU | 1.015(0.928-1.111) | 0.743 |  |  |
| NEU% | 1.009(0.985-1.035) | 0.460 |  |  |
| LYM% | 0.958(0.925-0.992) | 0.016 |  |  |
| LYM | 0.587(0.255-1.355) | 0.212 |  |  |
| MON% | 1.069(1.016-1.125) | 0.010 |  |  |
| MON | 1.803(0.819-3.972) | 0.143 |  |  |
| EOS% | 2.988(1.693-5.272) | 0.000 | 3.215(1.543-6.699) | 0.002 |
| EOS | 294.048(3.011-28720.250) | 0.015 |  |  |
| BAS% | 3.683(1.426-9.511) | 0.007 | 2.290(1.156-4.535) | 0.017 |
| BAS | 26.223(0.049-13978.578) | 0.308 |  |  |
| RBC | 1.048(0.532-2.064) | 0.892 |  |  |
| HGB | 1.011(0.985-1.037) | 0.413 |  |  |
| PLT | 0.964(0.944-0.984) | 0.001 |  |  |
| MPV | 0.758(0.625-0.918) | 0.005 |  |  |
| LDH | 1.001(1.001-1.002) | 0.000 |  |  |
| CK | 1.000(1.000-1.001) | 0.001 |  |  |
| ALT | 1.003(1.001-1.006) | 0.019 |  |  |
| AST | 1.004(1.003-1.006) | 0.000 | 1.003(1.001-1.005) | 0.001 |
| TBIL | 1.123(1.056-1.194) | 0.000 |  |  |
| DBIL | 1.195(1.095-1.305) | 0.000 | 1.120(1.004-1.248) | 0.041 |
| GGT | 1.006(1.003-1.010) | 0.000 |  |  |
| ALP | 1.017(1.008-1.026) | 0.000 |  |  |
| ALB | 0.792(0.710-0.883) | 0.000 |  |  |
| GLOB | 1.047(0.958-1.144) | 0.312 |  |  |
| GLU | 1.069(0.948-1.205) | 0.278 |  |  |
| UREA | 1.031(0.986-1.077) | 0.176 |  |  |
| CREA | 1.007(1.000-1.013) | 0.044 |  |  |
| K^+^ | 1.060(0.434-2.588) | 0.899 |  |  |
| NA^+^ | 1.008(0.951-1.068) | 0.793 |  |  |
| CA^2+^ | 0.015(0.001-0.334) | 0.008 |  |  |
| TT | 1.054(1.004-1.106) | 0.032 |  |  |
| APTT | 1.052(1.023-1.082) | 0.000 |  |  |
| PT | 1.003(0.993-1.013) | 0.547 |  |  |
| INR | 0.241(0.006-9.470) | 0.448 |  |  |
| PCT | 1.076(0.948-1.222) | 0.258 |  |  |
| CRP | 1.057(1.024-1.091) | 0.001 |  |  |

Abbreviations: CHD: Coronary Heart Disease, WBC: White Blood Cell, NEU: Neutrophil, LYM: Lymphocyte, MON: Monocyte, EOS: Eosinophils, BAS: Basophils, RBC: Red Blood Cell, HGB: Hemoglobin, PLT: Platelet, MPV: Mean Platelet Volume, LDH: Lactate dehydrogenase, CK: Creatine phosphokinase, ALT: Alanine aminotransaminase, AST: Aspartate aminotransferase, TBIL: Total Bilirubin, DBIL: Direct Bilirubin, GGT: γ-glutamyl transferase, ALP: Alkaline phosphatase, ALB: Albumin, GLOB: Globulin, GLU: Glucose, CREA: Creatinine, TT: Thrombin Time, APTT: Activated Partial Thromboplastin Time, PT: Prothrombin time, INR: Internationally Standardized Ratio, PCT: Procalcitonin, CRP: C-reactive protein, OR: Odds Ratio, CI: 95% Confidence Interval.
