# Supplementary material for: Eosinophils and basophils in severe fever with thrombocytopenia syndrome patients: Risk factors for predicting the prognosis on admission
Source: PLoS Negl Trop Dis. 2022 Dec 21;16(12):e0010967. doi: 10.1371/journal.pntd.0010967 (PMC9770358; doi:10.1371/journal.pntd.0010967)
Supplement: S2 Table — (DOCX) [file pntd.0010967.s003.docx]

**S2 Table. The predictive value of EOS%+BAS% for the prognosis on admission**

|  | **EOS%+BAS%** | | |
| --- | --- | --- | --- |
|  | **OR** | **95% CI** | ***P* value** |
| Model 1 | 282.867 | 19.263 – 4153.781 | 0.000 |
| Model 2 | 315.950 | 20.253 – 4928.774 | 0.000 |
| Model 3 | 227.224 | 14.235 – 3627.032 | 0.000 |
| Model 4 | 244.484 | 14.254 – 4193.356 | 0.000 |

Model 1: crude, no adjustment

Model 2: adjusting for age and gender.

Model3: adjusting for age and gender, temperature, arthralgia, hemorrhage, digestive symptoms, neurological symptoms and signs.

Model4: adjusting for age and gender, temperature, arthralgia, hemorrhage, digestive symptoms, neurological symptoms and signs, hypertensive disease and Coronary Heart Disease.

OR: Odds Ratio, CI: 95% Confidence Interval.
