# Supplementary material for: Eosinophils and basophils in severe fever with thrombocytopenia syndrome patients: Risk factors for predicting the prognosis on admission
Source: PLoS Negl Trop Dis. 2022 Dec 21;16(12):e0010967. doi: 10.1371/journal.pntd.0010967 (PMC9770358; doi:10.1371/journal.pntd.0010967)
Supplement: S3 Table — (DOCX) [file pntd.0010967.s004.docx]

**S3 Table. Differences between AUC of EOS%+BAS% and other factors AUC**

|  | **Difference of AUC** | **95% CI** | ***P* value** |
| --- | --- | --- | --- |
| EOS%+BAS% vs EOS% | 0.084 | -0.189 - 0.020 | 0.113 |
| EOS%+BAS% vs BAS% | 0.107 | -0.194 - 0.020 | 0.016 |
| EOS%+BAS% vs AST | 0.023 | -0.177 - 0.130 | 0.766 |
| EOS%+BAS% vs DBIL | 0.051 | -0.185 - 0.083 | 0.456 |
| EOS%+BAS% vs NLR | 0.217 | 0.048 - 0.386 | 0.012 |
| EOS%+BAS% vs De Ritis Ratio | 0.053 | -0.084 - 0.189 | 0.449 |

Abbreviations: EOS: Eosinophils, BAS: Basophil, AST: Aspartate aminotransferase, DBIL: Direct Bilirubin, AUC: Area Under the ROC Curve, CI: 95% Confidence Interval, De Ritis Ratio: AST/ALT-Ratio, NLR: Neutrophil-to-Lymphocyte Ratio.

D-value: Difference of Area Under the ROC Curve
