# Supplementary material for: Eosinophils and basophils in severe fever with thrombocytopenia syndrome patients: Risk factors for predicting the prognosis on admission
Source: PLoS Negl Trop Dis. 2022 Dec 21;16(12):e0010967. doi: 10.1371/journal.pntd.0010967 (PMC9770358; doi:10.1371/journal.pntd.0010967)
Supplement: S4 Table — (DOCX) [file pntd.0010967.s005.docx]

**S4 Table. Clinical characteristics of patients with SFTS, according to the EOS level on admission.**

| **Parameters** |  | **Groups** | | | ***P* value** | | | |
| --- | --- | --- | --- | --- | --- | --- | --- | --- |
|  | **Total (n=194)** | **A)EOS＜0.02 (n=164)** | **B)EOS = 0.02-0.5 (n=26)** | **C)EOS＞0.5**  **(n=4)** | **A vs B** | **B vs C** | **A vs C** | **A vs B vs C** |
| Clinic outcome, n (%) | 23/194(11.84) | 14/164(8.5) | 6/26(23.1) | 3/4(75.0) | 0.057 | 0.128 | 0.003 | 0.000 |
| Age, years | 62.39±11.85 | 61.98±11.21 | 64.04±13.98 | 68.75±22.14 | 0.401 | 0.565 | 0.584 | 0.000 |
| ≤45, n (%) | 16/194(8.2) | 14/164(8.5) | 1/26(3.8) | 1/4(25.0) | 0.665 | 0.253 | 0.315 | 0.906 |
| 46-60, n (%) | 64/194(33.0) | 55/164(33.5) | 9/26(34.6) | 0 | 0.914 | 0.388 | 0.383 | 0.489 |
| 61-75, n (%) | 86/194(44.3) | 76/164(46.3) | 9/26(34.6.0) | 1/4(25.0) | 0.264 | 1.000 | 0.735 | 0.211 |
| ≥76, n (%) | 28/194(14.4) | 19/164(11.6) | 7/26(26.9) | 2/4(50.0) | 0.035 | 0.725 | 0.077 | 0.003 |
| Male, n (%) | 101/194(52.1) | 88/164(53.7) | 11/26(42.3) | 2/4(50.0) | 0.282 | 1.000 | 1.000 | 0.369 |
| Time from onset to admission, days | 5.0(4.0-7.0) | 5.0(4.0-7.0) | 8.0(5.0-10.0) | 4.0(3.0-6.0) | 0.001 | 0.057 | 0.427 | 0.002 |
| ≤3, n (%) | 42/194(21.6) | 37/164(22.6) | 3/26(11.5) | 2/4(50.0) | 0.200 | 0.119 | 0.230 | 0.084 |
| 4-7, n (%) | 114/194(58.8) | 103/164(39.0) | 9/26(34.6) | 2/4(50.0) | 0.660 | 0.970 | 1.000 | 0.951 |
| ＞7, n (%) | 38/194(19.6) | 24/164(14.) | 14/26(53.8) | 0 | 0.000 | 0.141 | 0.537 | 0.002 |
| Hospitalization, days | 10.0(6.0-13.0) | 10.0(6.0-13.0) | 9.0(4-12.0) | 2.5(2.0-3.0) | 0.117 | 0.014 | 0.001 | 0.002 |
| ≤7, n (%) | 67/194(34.5) | 52/164(31.7) | 11/26(42.3) | 4/4(100) | 0.286 | 0.107 | 0.020 | 0.011 |
| 8-14, n (%) | 93/194(47.9) | 80/164(48.8) | 13/26(50.0) | 0 | 0.908 | 0.181 | 0.155 | 0.273 |
| ＜14, n (%) | 34/194(17.5) | 32/164(19.5) | 2/26(7.7) | 0 | 0.236 | 0.747 | 0.426 | 0.084 |
| Highest body temperature, ℃ | 38.0(37.0-38.8) | 38.0(37.0-38.8) | 38.0(36.9-38.9) | 38.0(37.8-38.4) | 0.930 | 0.691 | 0.751 | 0.943 |
| 38-38.9℃, n (%) | 60/194(30.9) | 49/164(29.9) | 8/26(30.8) | 3/4(75.0) | 0.972 | 0.249 | 0.167 | 0.210 |
| ＞39℃, n (%) | 40/194(20.6) | 34/164(20.7) | 6/26(23.1) | 0 | 0.785 | 0.388 | 0.401 | 0.678 |
| Bite by ticks, n (%) | 40/194(20.6) | 37/164(22.6) | 3/26(11.5) | 0 | 0.200 | 0.640 | 0.366 | 0.072 |
| Neurological Symptoms, n (%) | 23/194(11.9) | 14/164(8.5) | 6/26(23.1) | 3/4(75.0) | 0.057 | 0.128 | 0.003 | 0.000 |
| Confusion, n (%) | 14/194(7.2) | 9/164(5.5) | 4/26(15.4) | 1/4(25.0) | 0.150 | 0.538 | 0.219 | 0.038 |
| [Delirium](javascript:;), n (%) | 1/194(0.5) | 0 | 1/26(3.8) | 0 | 0.137 | 1.000 | - | 0.158 |
| Stupor, n (%), | 5/194(2.6) | 3/164(1.8) | 1/26(3.8) | 1/4(25.0) | 0.448 | 0.253 | 0.093 | 0.065 |
| [Somnolence](javascript:;) , n (%) | 1/194(0.5) | 1/164(0.6) | 0 | 0 | 1.000 | - | 1.000 | 0.850 |
| Coma, n (%) | 2/194(1.0) | 1/164(0.6) | 0 | 1/4(25) | 1.000 | 0.133 | 0.047 | 0.059 |
| Neurological signs, n (%) | 25/194(12.9) | 16/164(9.8) | 6/26(23.1) | 3/4(75.0) | 0.101 | 0.128 | 0.005 | 0.001 |

Abbreviations: EOS: Eosinophils, BAS: Basophil.

Continuous variable data are presented as median (interquartile ranges, IQR). Classified variable date are presented as n/N (%), where N is the total number of patients with available data.
