# Supplementary material for: Eosinophils and basophils in severe fever with thrombocytopenia syndrome patients: Risk factors for predicting the prognosis on admission
Source: PLoS Negl Trop Dis. 2022 Dec 21;16(12):e0010967. doi: 10.1371/journal.pntd.0010967 (PMC9770358; doi:10.1371/journal.pntd.0010967)
Supplement: S5 Table — (DOCX) [file pntd.0010967.s006.docx]

**S5 Table. Clinical characteristics of patients with SFTS, according to the EOS whether decreased to Undetectable on admission.**

| **Parameters** | **Total (n=194)** | **EOS=0 (n=125)** | **EOS＞0 (n=69)** | ***P* value** |
| --- | --- | --- | --- | --- |
| Clinic outcome, n (%) | 23/194(11.84) | 11/125(8.8) | 12/69(17.4) | 0.076 |
| Age, years | 62.39±11.85 | 61.54±11.13 | 63.93±13.00 | 0.181 |
| ≤45, n (%) | 16/194(8.2) | 11/125(8.8) | 5/69(7.2) | 0.706 |
| 46-60, n (%) | 64/194(33.0) | 45/125(36.0) | 19/69(27.5) | 0.230 |
| 61-75, n (%) | 86/194(44.3) | 56/125(44.8) | 30/69(43.5) | 0.859 |
| ≥76, n (%) | 28/194(14.4) | 13/125(10.4) | 15/69(21.7) | 0.031 |
| Male, n (%) | 101/194(52.1) | 64/125(51.2) | 37/69(53.6) | 0.746 |
| Time from onset to admission, days | 5.0(4.0-7.0) | 5.00(4.00-6.00) | 5.00(4.00-8.00) | 0.059 |
| ≤3, n (%) | 42/194(21.6) | 28/125(22.4) | 14/69(20.3) | 0.733 |
| 4-7, n (%) | 114/194(58.8) | 80/125(64.0) | 34/69(49.3) | 0.046 |
| ＞7, n (%) | 38/194(19.6) | 17/125(13.6) | 21/69(30.4) | 0.005 |
| Hospitalization, days | 10.0(6.0-13.0) | 10.00(6.00-13.00) | 9.00(4.50-12.50) | 0.133 |
| ≤7, n (%) | 67/194(34.5) | 38/125(30.4) | 29/69(42.0) | 0.103 |
| 8-14, n (%) | 93/194(47.9) | 65/125(52.0) | 28/69(40.6) | 0.127 |
| ＞14, n (%) | 34/194(17.5) | 22/125(17.6) | 12/69(17.4) | 0.971 |
| Highest body temperature,℃ | 38.0(37.0-38.8) | 38.0(36.9-38.7) | 38.0(37.0-38.8) | 0.910 |
| 38-38.9℃, n (%) | 60/194(30.9) | 38/125(30.4) | 22/69(31.9) | 0.830 |
| ＞39℃, n (%) | 40/194(20.6) | 26/125(20.8) | 14/69(20.3) | 0.933 |
| Bite by ticks, n (%) | 40/194(20.6) | 30/125(24.0) | 10/69(14.5) | 0.117 |
| Neurological Symptoms, n (%) | 23/194(11.9) | 11/125(8.8) | 12/69(17.4) | 0.076 |
| Confusion, n (%) | 14/194(7.2) | 6/125(4.8) | 8/69(11.6) | 0.144 |
| [Delirium](javascript:;) , n (%) | 1/194(0.5) | 0 | 1/69(1.4) | 0.356 |
| Stupor, n (%) | 5/194(2.6) | 3/125(2.4) | 2/69(2.9) | 1.000 |
| [Somnolence](javascript:;), n (%) | 1/194(0.5) | 1/125(0.8) | 0 | 1.000 |
| coma, n (%) | 2/194(1.0) | 11/25(0.8) | 1/69(1.4) | 1.000 |
| Neurological signs, n (%) | 25/194(12.9) | 12/125(9.6) | 13/69(18.8) | 0.066 |

Abbreviations: EOS: Eosinophils, BAS: Basophil.

Continuous variable data are presented as median (interquartile ranges, IQR).

Classified variable date are presented as n/N (%), where N is the total number of patients with available data. *P* values comparing between the group of EOS=0 and the group of EOS＞0.
