# Supplementary material for: Eosinophils and basophils in severe fever with thrombocytopenia syndrome patients: Risk factors for predicting the prognosis on admission
Source: PLoS Negl Trop Dis. 2022 Dec 21;16(12):e0010967. doi: 10.1371/journal.pntd.0010967 (PMC9770358; doi:10.1371/journal.pntd.0010967)
Supplement: S6 Table — (DOCX) [file pntd.0010967.s007.docx]

**S6 Table. Correlation between circulating EOS%, BAS% and neurological manifestations in SFTS patients.**

| **Parameters** | **EOS%** | | **BAS%** | |
| --- | --- | --- | --- | --- |
|  | **r** | ***P*** | **r** | ***P*** |
| Neurological symptoms | 0.158 | 0.028 | 0.109 | 0.131 |
| Confusion | 0.156 | 0.030 | 0.083 | 0.247 |
| Delirium | 0.132 | 0.067 | 0.025 | 0.726 |
| Stupor | -0.007 | 0.921 | -0.085 | 0.238 |
| Somnolence | 0.071 | 0.325 | 0.121 | 0.092 |
| Coma | -0.025 | 0.725 | 0.164 | 0.023 |
| Neurological signs | 0.180 | 0.012 | 0.146 | 0.043 |

Abbreviations: EOS: Eosinophils, BAS: Basophil.
