# Supplementary material for: Eosinophils and basophils in severe fever with thrombocytopenia syndrome patients: Risk factors for predicting the prognosis on admission
Source: PLoS Negl Trop Dis. 2022 Dec 21;16(12):e0010967. doi: 10.1371/journal.pntd.0010967 (PMC9770358; doi:10.1371/journal.pntd.0010967)
Supplement: S7 Table — (DOCX) [file pntd.0010967.s008.docx]

**S7 Table. Clinical characteristics of patients with SFTS, according to the BAS level on admission.**

| **Parameters** | **Total (n=194)** | **BAS＜0.06(n=186)** | **BAS≥0.06(n=8)** | ***P* value** |
| --- | --- | --- | --- | --- |
| Clinic outcome, n (%) | 23/194(11.84) | 19/186(10.1) | 4/8(50.0) | 0.007 |
| Age, years | 62.39±11.85 | 62.13±11.89 | 68.38±9.47 | 0.145 |
| ≤45, n (%) | 16/194(8.2) | 16/194(8.2) | 0 | 0.496 |
| 46-60, n (%) | 64/194(33.0) | 64/194(33.0) | 1/8(12.5) | 0.382 |
| 61-75, n (%) | 86/194(44.3) | 86/194(44.3) | 6/8(75.0) | 0.156 |
| ≥76, n (%) | 28/194(14.4) | 28/194(14.4) | 1/8(12.5) | 1.000 |
| Male, n (%) | 101/194(52.1) | 96/186(51.) | 5/8(62.5) | 0.809 |
| Time from onset to admission, days | 5.0(4.0-7.0) | 5.0(4.0-7.0) | 6.5(4.5-8.8) | 0.170 |
| ≤3, n (%) | 42/194(21.6) | 41/186(22.0%) | 1/8(12.5) | 0.839 |
| 4-7, n (%) | 114/194(58.8) | 110/186(59.1) | 4/8(50.0) | 0.883 |
| ＞7, n (%) | 38/194(19.6) | 35/186(18.8) | 3/8(37.5) | 0.396 |
| Hospitalization, days | 10.0(6.0-13.0) | 10.0(6.0-13.0) | 8.0(2.0-12.5) | 0.215 |
| ≤7, n (%) | 67/194(34.5) | 63/186(33.9) | 4/8(50.0) | 0.576 |
| 8-14, n (%) | 93/194(47.9) | 89/186(47.8) | 4/8(50.0) | 1.000 |
| ＞14, n (%) | 34/194(17.5) | 34/186(18.3) | 0 | 0.392 |
| Highest body temperature, ℃ | 38.0(37.0-38.8) | 38.0(37.0-38.8) | 37.9(37.0-38.8) | 0.979 |
| 38-38.9℃, n (%) | 60/194(30.9) | 57/186(30.6) | 3/8(37.5) | 0.984 |
| ＞39℃, n (%) | 40/194(20.6) | 26/186(14.0) | 1/8(12.5) | 1.000 |
| Bite by ticks, n (%) | 40/194(20.6) | 40/186(21.5) | 0 | 0.305 |
| Neurological Symptoms, n (%) | 23/194(11.9) | 19/186(10.2) | 4/8(50.0) | 0.008 |
| Confusion, n (%) | 14/194(7.2) | 13/186(7.0) | 1/8(12.5) | 0.457 |
| [Delirium](javascript:;) , n (%) | 1/194(0.5) | 1/186(0.5) | 0 | 1.000 |
| Stupor, n (%) | 5/194(2.6) | 5/186(2.7) | 1/8(12.5) | 0.226 |
| [Somnolence](javascript:;), n (%) | 1/194(0.5) | 0 | 2/8(25.0) | 0.001 |
| Coma, n (%) | 2/194(1.0) | 0 | 2/8(25.0) | 0.001 |
| Neurological signs, n (%) | 25/194(12.9) | 21/186(11.3) | 4/8(50.0) | 0.008 |

Abbreviations: EOS: Eosinophils, BAS: Basophil.

Continuous variable data are presented as median (interquartile ranges, IQR).

Classified variable date are presented as n/N (%), where N is the total number of patients with available data. *P* values comparing between the group of BAS ＜0.06 and the group of BAS≥0.06.
