# Supplementary material for: Eosinophils and basophils in severe fever with thrombocytopenia syndrome patients: Risk factors for predicting the prognosis on admission
Source: PLoS Negl Trop Dis. 2022 Dec 21;16(12):e0010967. doi: 10.1371/journal.pntd.0010967 (PMC9770358; doi:10.1371/journal.pntd.0010967)
Supplement: S8 Table — (DOCX) [file pntd.0010967.s009.docx]

**S8 Table. Correlation between circulating EOS%、BAS% and laboratory paraments of SFTS patients.**

| **Parameters** | **EOS%** | | **BAS%** | |
| --- | --- | --- | --- | --- |
|  | **r** | ***P*** | **r** | ***P*** |
| NEU% | -0.129 | 0.073 | -0.142 | 0.048 |
| LYM% | 0.045 | 0.536 | -0.018 | 0.804 |
| MON% | 0.084 | 0.245 | 0.292 | 0.000 |
| EOS% | / | / | 0.308 | 0.000 |
| PLT | 0.066 | 0.357 | -0.092 | 0.200 |
| CRP | 0.058 | 0.421 | 0.143 | 0.047 |
| PCT | 0.089 | 0.217 | -0.071 | 0.328 |
| LDH | 0.011 | 0.884 | 0.299 | 0.000 |
| CK | -0.012 | 0.837 | 0.216 | 0.003 |
| ALT | 0.029 | 0.688 | 0.185 | 0.010 |
| AST | 0.047 | 0.517 | 0.189 | 0.008 |
| TBIL | 0.183 | 0.011 | 0.220 | 0.002 |
| DBIL | 0.183 | 0.011 | 0.284 | 0.000 |
| ALB | -0.196 | 0.006 | -0.292 | 0.000 |
| GGT | 0.115 | 0.109 | 0.312 | 0.000 |
| ALP | 0.146 | 0.042 | 0.247 | 0.001 |
| UREA | 0.117 | 0.105 | 0.170 | 0.018 |
| CREA | 0.027 | 0.714 | 0.169 | 0.018 |
| PT | -0.050 | 0.487 | -0.237 | 0.001 |

Abbreviations: EOS: Eosinophils, BAS: Basophil, NEU: Neutrophil, LYM: Lymphocyte, MON: Monocyte, PLT: Platelet, CRP: C-reactive protein, PCT: Procalcitonin, LDH: Lactate dehydrogenase, CK: Creatine phosphokinase, ALT: Alanine aminotransaminase, AST: Aspartate aminotransferase, TBIL: Total Bilirubin, DBIL: Direct Bilirubin, ALB: Albumin, GGT: γ-glutamyl transferase, ALP: Alkaline phosphatase, CREA: Creatinine, PT: Prothrombin time.
